# Symbiodiniaceae Are the First Site of Heterotrophic Nitrogen Assimilation in Reef-Building Corals

**DOI:** 10.1128/mbio.01601-22

**Published:** 2022-09-20

**Authors:** Stephane Martinez, Renaud Grover, David M. Baker, Christine Ferrier-Pagès

**Affiliations:** a Coral Ecophysiology Team, Centre Scientifique de Monaco, Monaco, Monaco; b The Swire Institute of Marine Science, School of Biological Sciences, The University of Hong Kong, Hong Kong, People’s Republic of China; Carnegie Institution for Science; University of Hawaii at Manoa

**Keywords:** pulse-chase, stable isotopes, coral reefs, nitrogen, Symbiodiniaceae, compound-specific isotope analysis of amino acids, dinoflagellate, heterotrophy

## Abstract

Coral reefs depend on the highly optimized mutualistic relationship between corals and Symbiodiniaceae dinoflagellates. Both partners exchange nutrients obtained through heterotrophy of the host and autotrophy of the symbionts. While heterotrophy helps corals withstand the harmful effects of seawater warming, the exchange of heterotrophic nutrients between the two partners is poorly understood. Here, we used compound-specific *δ*^15^N and *δ*^13^C of amino acids (*δ*^15^N_AA_ and *δ*^13^C_AA_) and a ^15^N pulse-chase experiment with Artemia salina nauplii in two coral-dinoflagellate associations to trace the assimilation and allocation of heterotrophic nutrients within the partners. We observed that changes in the trophic position (TP_Glx-Phe_), *δ*^15^N_AA_, and *δ*^13^C_AA_ with heterotrophy were holobiont-dependent. Furthermore, while TP_Glx-Phe_ and *δ*^15^N of all AAs significantly increased with heterotrophy in the symbionts and host of Stylophora pistillata, only the *δ*^15^N_AA_ of the symbionts changed in Turbinaria reniformis. Together with the pulse-chase experiment, the results suggested a direct transfer of heterotrophically acquired AAs to the symbionts of S. pistillata and a transfer of ammonium to the symbionts of T. reniformis. Overall, we demonstrated that heterotrophy underpinned the nutrition of Symbiodinaceae and possibly influenced their stress tolerance under changing environmental conditions.

## INTRODUCTION

Symbiosis with photosynthetic dinoflagellates belonging to Symbiodiniaceae (1) is observed in many cnidarians and has contributed to the rapid adaptation of these organisms to a wide range of environmental conditions (2). Symbiodiniaceae and reef-building corals have developed tight metabolic and nutritional interactions that are essential for coral survival in nutrient-poor waters. Algal symbionts perform photosynthesis and assimilate dissolved inorganic nitrogen (N) and phosphorus (P) (3–5). Symbiont photosynthates, mainly sugars but also lipids and amino acids, largely support coral metabolism and calcification (6, 7). In return, the host provides the algae with nutrients derived from its catabolism (8) or acquired from its heterotrophic feeding on planktonic prey and detritus (9). This mutual exchange of metabolites between the symbiotic partners forms the basis of the coral reef ecosystem.

Under the characteristic oligotrophic conditions of coral reefs, dissolved inorganic N and P availability is limited (10). Therefore, many corals rely on heterotrophy as an additional source of essential nutrients (11, 12). However, there is still a lack of tools to distinguish the autotrophic and heterotrophic origins of nutrients (13). Therefore, the extent to which coral species rely on heterotrophic diets under different environmental conditions is still poorly understood. Nevertheless, in laboratory experiments, heterotrophy is highly beneficial to corals, promoting photosynthesis and calcification (14). Most importantly, it strengthens coral resistance to heat stress, ocean acidification, or pathogen infections (15–17). Heterotrophy mitigates bleaching (13, 17), accelerates postbleaching recovery, and reduces mortality (15, 18). The transfer of metals present in the heterotrophic diet to symbionts during stress is key to the success of the symbiosis in supporting symbiont growth and maintenance (17). Symbionts are also limited in nitrogen in the host tissue (19–22), so access to heterotrophic nitrogen may be critical for their stress resistance. However, knowledge of the translocation of heterotrophic nitrogen from the host to its algal symbionts is still limited (15, 17). Although a few studies have shown that heterotrophic nitrogen was exchanged between host and symbionts, this was mainly achieved through measurements of bulk ^15^N in tissue samples (9, 23–25), and the nature of the nitrogen assimilated in symbionts (heterotrophic amino acids, recycled ammonium, etc.), has not been directly investigated. Nevertheless, Piniak and Lipschultz (24) argued that the presence of heterotrophic N in symbionts within 4 h was insufficient time for complete recycling of N from host metabolism and that symbionts may directly assimilate dietary amino acids. Therefore, further studies are needed to fully understand the percentage of translocated nitrogen and the nature of nitrogen compounds exchanged between the coral host and symbionts. Efforts should also be made to find the best proxy to trace heterotrophy under *in situ* conditions and understand its importance for reef corals.

**FIG 1:**
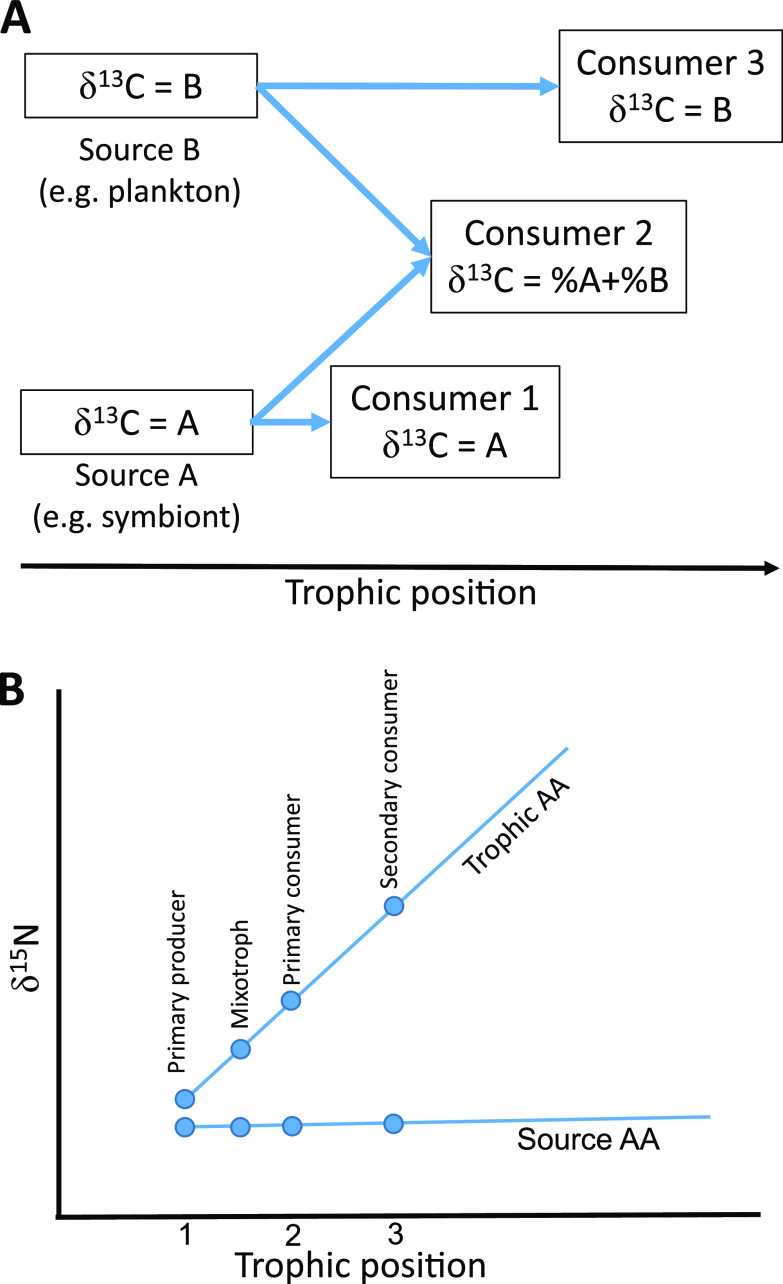
Schematic explanation of changes in carbon and nitrogen isotope values of amino acids (AA) along the food chain. (A) Each food source has its carbon isotopic signature of essential AA. A consumer feeding exclusively on one source had the same essential AA carbon signature. A consumer who fed on two (or more) food sources had their combined AA signature, depending on the proportion of the diet they provided. (B) When the trophic position increases, the nitrogen isotope value of the source AA (i.e., phenylalanine) remained unchanged. In contrast, the nitrogen isotope value of the trophic AA (i.e., glutamic acid) increased (became more positive) with each change in trophic position.

Compound-specific isotope analysis (CSIA) of amino acids (AAs) is a powerful tool for obtaining information about an organism's food sources and understanding trophic interactions in mixotrophic symbioses (26). In theory, animals cannot synthesize essential AAs (AA_ess_) *de novo* and must acquire them from the diet. Moreover, taxa that can synthesize AA_ess_ (e.g., bacteria, fungi, microalgae, or macroalgae) usually present a unique δ^13^C fingerprint, with little to no δ^13^C-AA_ess_ isotopic fractionation between predators and prey (Fig. 1A) (27). Therefore, their ingestion by the host can be traced by comparing the host-prey δ^13^C-AA_ess_. In turn, nitrogen (δ^15^N) enrichment of “trophic AAs” (such as glutamic acid) occurs in the animal compared with the same AAs in the diet. In contrast, source AAs (such as phenylalanine) show very little enrichment (Fig. 1B) (28, 29). Therefore, regardless of the isoscape, the δ^15^N difference between glutamic acid and phenylalanine was used to calculate the trophic position of an organism (TP_Glx-Phe_), i.e., the level it occupies in a food web (28, 30). A TP_Glx-Phe_ of 1 represents autotrophs, whereas higher TP_Glx-Phe_ values represent primary and secondary consumers (28).

Although the above “isotopic” rules apply to most heterotrophic animals, they cannot be systematically used in the Symbiodiniaceae-coral symbiosis because the two partners are interrelated and have evolved unique adaptations for the symbiosis. Furthermore, the origin of AA_ess_ in coral holobionts is blurred by the fact that, unlike most metazoans, some coral hosts, but not all, have retained the ability to synthesize AA_ess_ (8, 31–33). Because they also have tight recycling of elements with their symbionts, heterotrophically fed corals may present different or similar δ^13^C-AA_ess_ values than autotrophic corals, depending on the species or diet type (34–36). Similarly, the TP_Glx-Phe_ index of heterotrophically fed corals may be higher or identical to that of autotrophic colonies (35, 36). Only a few studies have applied CSIA-AA to trace the nutritional ecology of corals (11, 34–38). While Fox et al. (34) found highly differentiated autotrophic and heterotrophic *δ*^13^C-AAs fingerprints in some coral holobionts, only a limited effect of heterotrophy on *δ*^13^C-AA_s_ was measured in other coral holobionts (11, 35, 36). Such differences could be related to the coral-Symbiodiniaceae association studied or to the quality and quantity of food provided to the symbiotic associations. Similarly, Wall et al. (36) observed a consistent overlap in *δ*^15^N_AA_ values of hosts and symbionts, while Ferrier-Pagès et al. (35) measured different *δ*^15^N values of glutamic acid and phenylalanine in host and symbionts when corals were supplied with heterotrophic diet. Thus, these observations strongly support the necessity of more studies on CSIA-AAs in corals. Indeed, understanding nutrient fluxes between partners is crucial to uncovering the role of heterotrophy in the success of coral-dinoflagellate symbioses exposed to environmental stress.

To resolve the isotopic fingerprint of heterotrophy, we measured in a first experiment the *δ*^13^C and *δ*^15^N values of a broad spectrum of AAs in two widespread Red Sea coral holobionts (Stylophora pistillata and Turbinaria reniformis associated with *Symbiodinium* clade A1 and *Cladocopium* clade C1). For several weeks, corals were maintained in three feeding regimes (autotrophy, mixotrophy, and heterotrophy) or analyzed immediately after sampling. This work followed a preliminary study conducted only on S. pistillata (35) and for two amino acids (glutamic acid and phenylalanine). This experiment addressed three main questions. First, do we observe a significant change in host tissue *δ*^13^C and *δ*^15^N-AAs levels when corals are fed a heterotrophic diet? If the dietary amino acids are assimilated into the host tissue, we sought to understand which dietary amino acids are retained directly and whether the same changes occur in the two symbiotic associations. Second, is there translocation of dietary amino acids from the host to the symbionts? If so, which amino acids are preferentially translocated, and do we observe the same pattern in both symbioses? Third, can we compare the *δ*^13^C and *δ*^15^N-AAs levels of symbiotic associations between laboratory and reef conditions?

In a second experiment, we fed corals with ^15^N-labeled Artemia salina and performed a pulse-chase experiment in which we sampled corals at 12 h, 36 h, 72 h, and 168 h after feeding. The main question of this second experiment was how fast ^15^N-AAs were transferred from the host to the symbionts and whether the nitrogen remained in or was lost from the symbiotic association after feeding. The results obtained provided a better understanding of the nutrient exchange between the partners of a symbiotic association. Furthermore, they suggested that the transfer of heterotrophic nitrogen from the host to the symbionts was a crucial process leading to stable and specific cnidarian-host-Symbiodiniaceae partnerships.

## RESULTS

### Trophic experiment.

**FIG 2:**
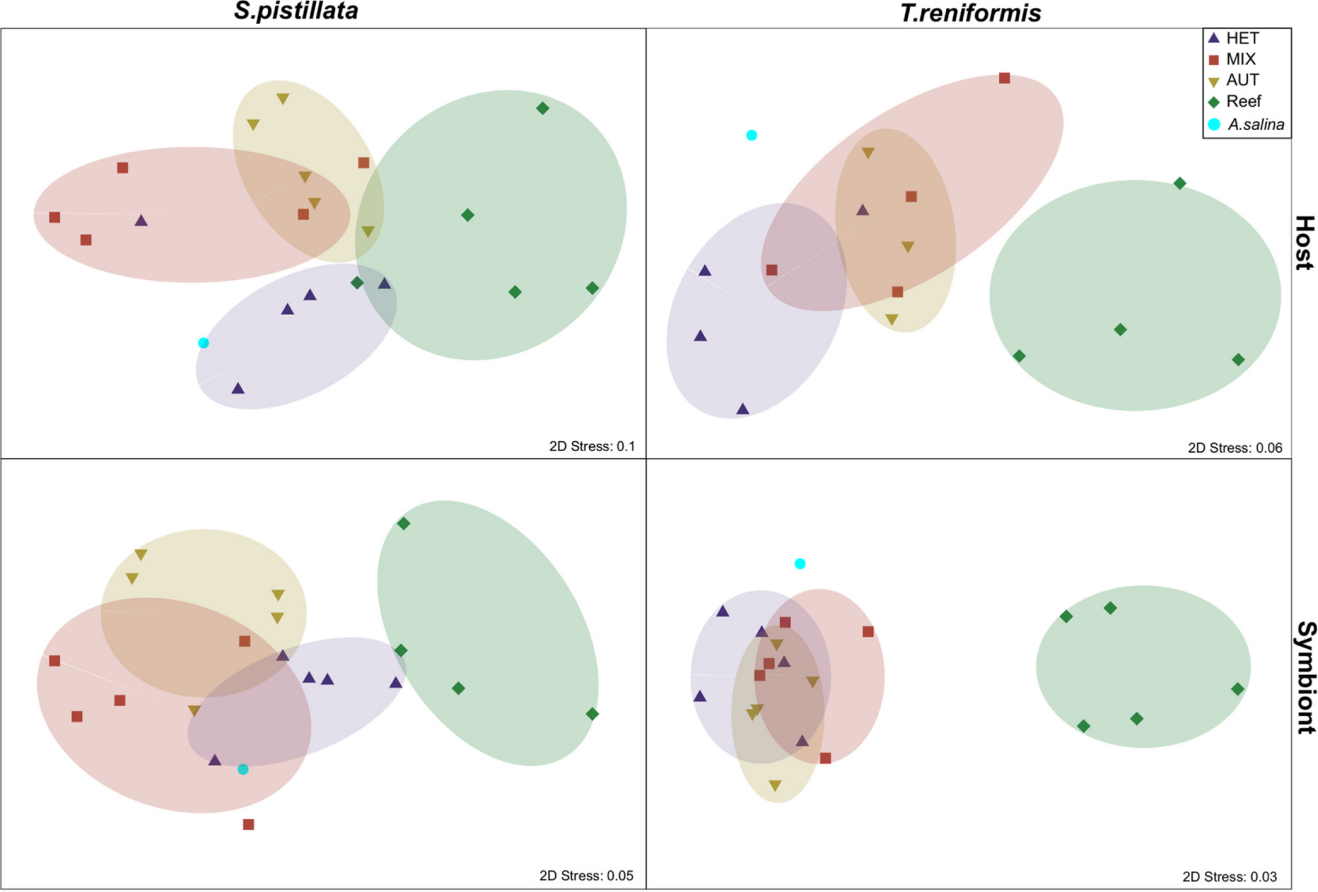
nMDS of the δ^13^C-AAs (valine, leucine, isoleucine, methionine, and phenylalanine) for S. pistillata and T. reniformis host tissue and algal symbionts in the different treatments. The treatments were heterotrophic (HET, purple triangle), mixotrophic (MIX, red square), autotrophic (AUT, yellow triangle), natural reef (Reef, green diamond), and Artemia salina nauplii (A. salina, cyan square).

Nonmetric multidimensional scaling (NMDS) plots based on all values of *δ*^13^C-AAs (valine, leucine, isoleucine, methionine, and phenylalanine) of tissue and symbiont samples of S. pistillata and T. reniformis were created, using the Euclidean distance matrices. Axes define 2D space that allowed the best spatial representation of sample distance based on Euclidean distance with stress between 0.03 and 0.1. Ellipses divide the data based on nutritional treatment. Because nMDS relies on rank orders for ordination, the closeness of data points indicated how similar they were. Nonoverlapping centroids were considered significantly different at α = 0.05. In S. pistillata, there was no significant difference between host and symbiont δ^13^C-AAs because the ellipses overlapped (*P* = 0.67, Fig. S1). However, there was an overall significant difference between feeding treatments (*P* < 0.01 and *P* < 0.01 for host and symbionts, Fig. 2) and with the reef samples (*P* < 0.03, Fig. 2) as shown by the separation of the ellipses on the nMDS. The significant difference was between the heterotrophy (HET) and autotrophy (AUT) conditions for the host and symbionts (*P* < 0.03) and between the mixotrophic (MIX) and AUT for the symbionts (*P* = 0.03). In contrast to S. pistillata, there was a significant difference in δ^13^C-AAs between the host and symbionts of T. reniformis (*P* < 0.01), mainly in the MIX and AUT treatments (*P* = 0.04 and *P* = 0.03, respectively, Fig. S1). There was, however, no significant difference in the *δ*^13^C-AAs of the host and symbionts between the trophic conditions (Fig. 2, *P* > 0.09 and *P* > 0.25, respectively), except for the reef samples. The δ^13^C-AAs values of reef samples (Fig. S1) were significantly higher (more positive) than the HET and MIX treatments for the host (*P* = 0.03 and *P* = 0.02, respectively) and from all aquaria treatments for the symbionts (*P* < 0.01).

10.1128/mbio.01601-22.1 FIG S1 nMDS and comparison of the *δ*^13^C-AAs (valine, leucine, isoleucine, methionine, and phenylalanine) for S. pistillata and T. reniformis host tissue and algal symbionts in the different treatments. The treatments were heterotrophy (HET, blue), mixotrophy (MIX, red), autotrophy (AUT, yellow), and reef samples (Reef, green). The circle represents the symbionts, while the triangle represents the host. The circles in the nMDS represent a significant difference (*P* < 0.05) between the host and symbiont. Download FIG S1, EPS file, 0.4 MB. Copyright © 2022 Martinez et al. 2022 Martinez et al. https://creativecommons.org/licenses/by/4.0/ This content is distributed under the terms of the Creative Commons Attribution 4.0 International license.

**FIG 3:**
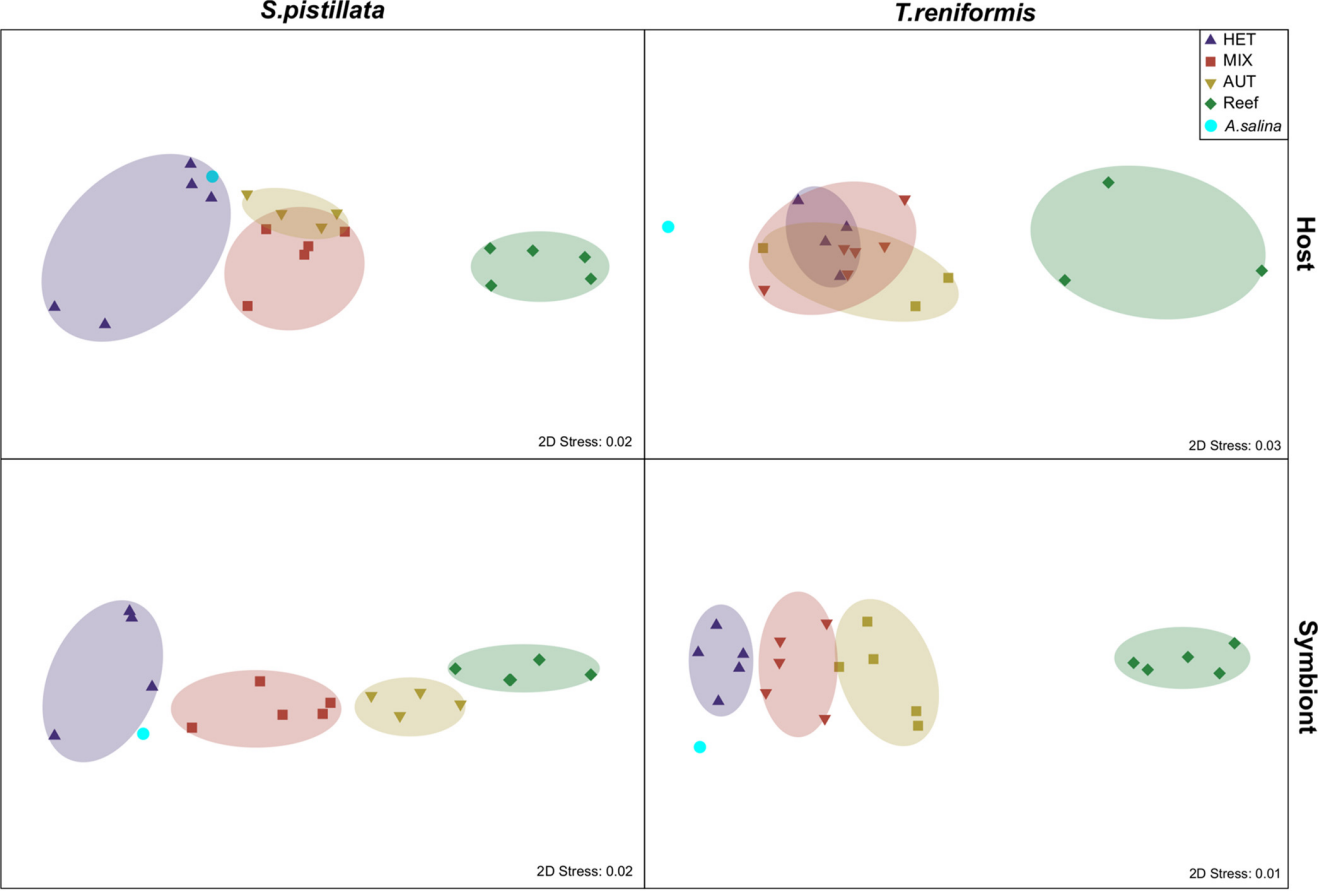
nMDS plot performed using the *δ*^15^N value of the amino acids (alanine, glycine, proline, aspartic-acid, glutamic-acid, valine, leucine, isoleucine, methionine, and phenylalanine) of the host and symbiont compartments of the corals S. pistillata and T. reniformis. The treatments are heterotrophic (HET, purple triangle), mixotrophic (MIX, red square), autotrophic (AUT, yellow triangle), natural reef (Reef, green diamond), and Artemia salina nauplii (A. salina, cyan square).

**FIG 4:**
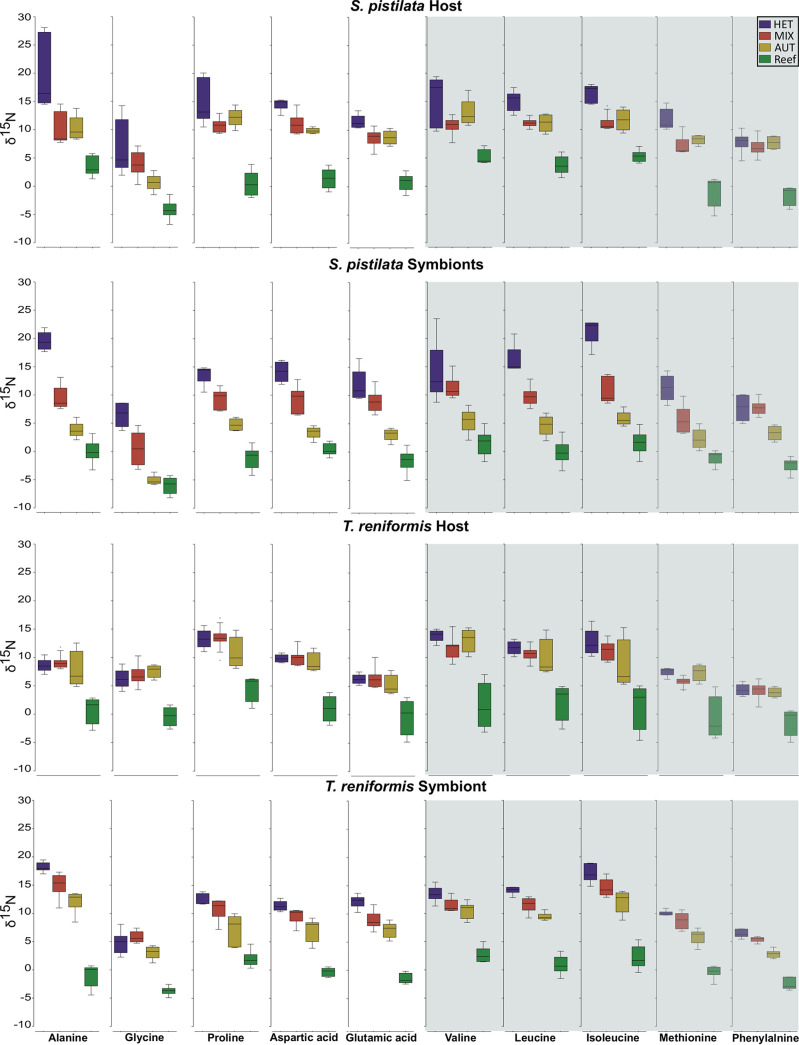
Comparison of the δ^15^N-AAs values of the host and symbionts of S. pistillata and T. reniformis. Amino acids with a white background are nonessential, and those with a gray background are essential AAs. The four treatments were heterotrophic (purple), mixotrophic (red), autotrophic (yellow), and natural reef (green).

**FIG 5:**
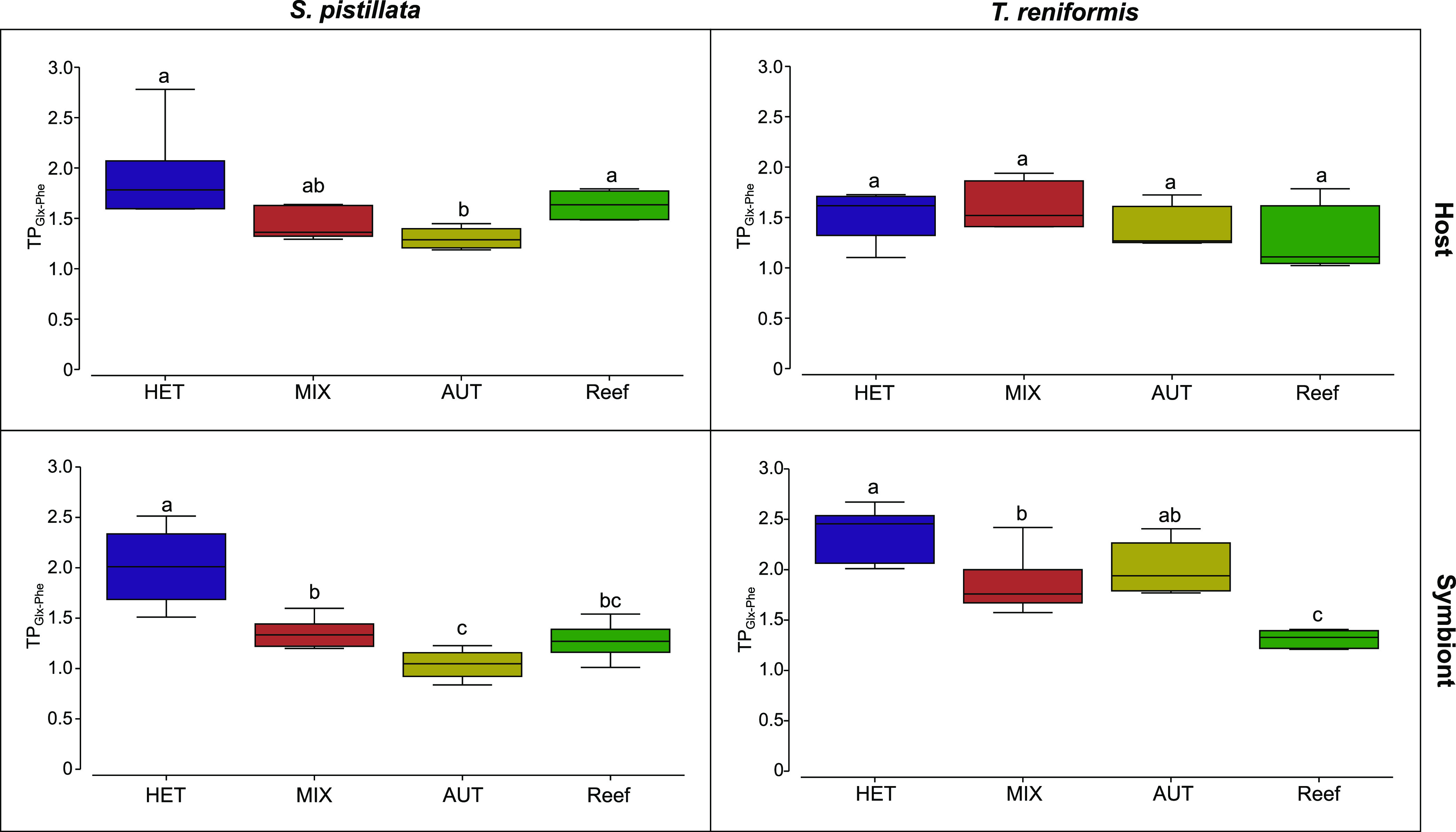
Trophic position index of the host and symbiont compartments of S. pistillata and T. reniformis. This index was calculated based on glutamic acid and phenylalanine *δ*^15^N values. The four treatments were heterotrophic (HET, purple), mixotrophic (MIX, red), autotrophic (AUT, yellow), and natural reef (Reef, green). The different letters represent a significant difference (*P* < 0.05) between treatments.

nMDS plots based on all values of *δ*^15^N-AAs of tissue and symbiont samples were also created, using the Euclidean distance matrices. Ellipses divide the data based on nutritional treatment. Concerning S. pistillata *δ*^15^N-AAs, there was a significant difference between the values of host and symbionts in the AUT group (*P* = 0.03, Fig. S2). Feeding treatments were also significantly different when there was a clear separation of the ellipses on the nMDS (*P* < 0.01, Fig. 3). For the host tissue, the *δ*^15^N-AAs values of the HET condition were closed to the artemia values (Fig. 3 and Table 1) and significantly different from the MIX and AUT *δ*^15^N-AAs values (*P* = 0.01 and *P* = 0.02, respectively, Fig. 3 and 4). All treatments were significantly different and well separated in the symbionts nMDS, with a decrease in *δ*^15^N-AAs values from the heterotrophic to the mixotrophic and autotrophic conditions (*P* < 0.03, Fig. 3 and 4). In addition, reef samples presented significantly lower δ^15^N-AAs values of host and symbionts than all other treatments (*P* < 0.01, Fig. 4). The TP_Glx-Phe_ of host and symbionts followed the *δ*^15^N-AAs patterns, with the lowest values in the autotrophic condition and the highest in the heterotrophic condition (Fig. 5). The TP_Glx-Phe_ of reef samples was similar to the TP_Glx-Phe_ of the HET and MIX samples in the host tissue (*P* = 0.24 and *P* = 0.1, respectively), and of MIX and AUT samples in the symbiont compartment (*P* = 0.53 and 0.1, respectively).

**TABLE 1:** Carbon and nitrogen isotope values (‰) of natural and ^15^N-labeled Artemia salina nauplii

Parameter	Alanine	Glycine	Valine	Leucine	Isoleucine	Threonine	Serine	Proline	Aspartic acid	Methionine	Glutamic acid	Phenylalanine
Labeled *δ*^15^N	14117	12311	13755	13956	12897	13847	12513	11976	15207	13979	13866	14888
Natural *δ*^15^N	15.9	3.0	17.3	16.1	15.0	NA^*a*^	NA	10.5	12.8	7.7	14.1	8.0
Natural *δ*^13^C	NA	NA	−30.5	−27.5	−21.1	NA	NA	NA	NA	−19.9	NA	−25.5

a NA, missing data.

10.1128/mbio.01601-22.2 FIG S2 nMDS of the δ^15^N-AAs (alanine, glycine, proline, aspartic-acid, glutamic-acid, valine, leucine, isoleucine, methionine, and phenylalanine) for S. pistillata and T. reniformis host tissue and algal symbionts in the different treatments. The treatments were heterotrophy (HET, blue), mixotrophy (MIX, red), autotrophy (AUT, yellow), and reef samples (Reef, green). The circle represents the symbionts, while the triangle represents the host. The circles in the nMDS represent a significant difference (*P* < 0.05) between the host and symbiont. Download FIG S2, EPS file, 0.2 MB. Copyright © 2022 Martinez et al. 2022 Martinez et al. https://creativecommons.org/licenses/by/4.0/ This content is distributed under the terms of the Creative Commons Attribution 4.0 International license.

For T. reniformis, there was a significant difference in *δ*^15^N-AAs between host and symbiont of the HET and MIX groups (*P* < 0.01, Fig. S2), as well as a significant difference in the *δ*^15^N-AAs between treatments (*P* < 0.01, Fig. 3). At the host level, only the *δ*^15^N-AAs values of the reef samples were significantly different from the aquaria treatments with lower values (*P* < 0.03, Fig. 3 and 4). The TP_Glx-Phe_ of the host (Fig. 5) was not significantly different between the experimental treatments or with the reef samples (*P* = 0.18). Contrary to the host tissue, the *δ*^15^N AAs values of the symbionts were all different among treatments (*P* < 0.02, Fig. 3) and decreased from HET to MIX, AUT, and reef conditions (Fig. 4). The TP_Glx-Phe_ of the symbionts was also significantly different between HET and MIX conditions (*P* = 0.04, Fig. 5). The reef samples had a significantly lower TP_Glx-Phe_ than all the aquaria treatments (*P* < 0.01, Fig. 5).

### Pulse-chase experiment.

**FIG 6:**
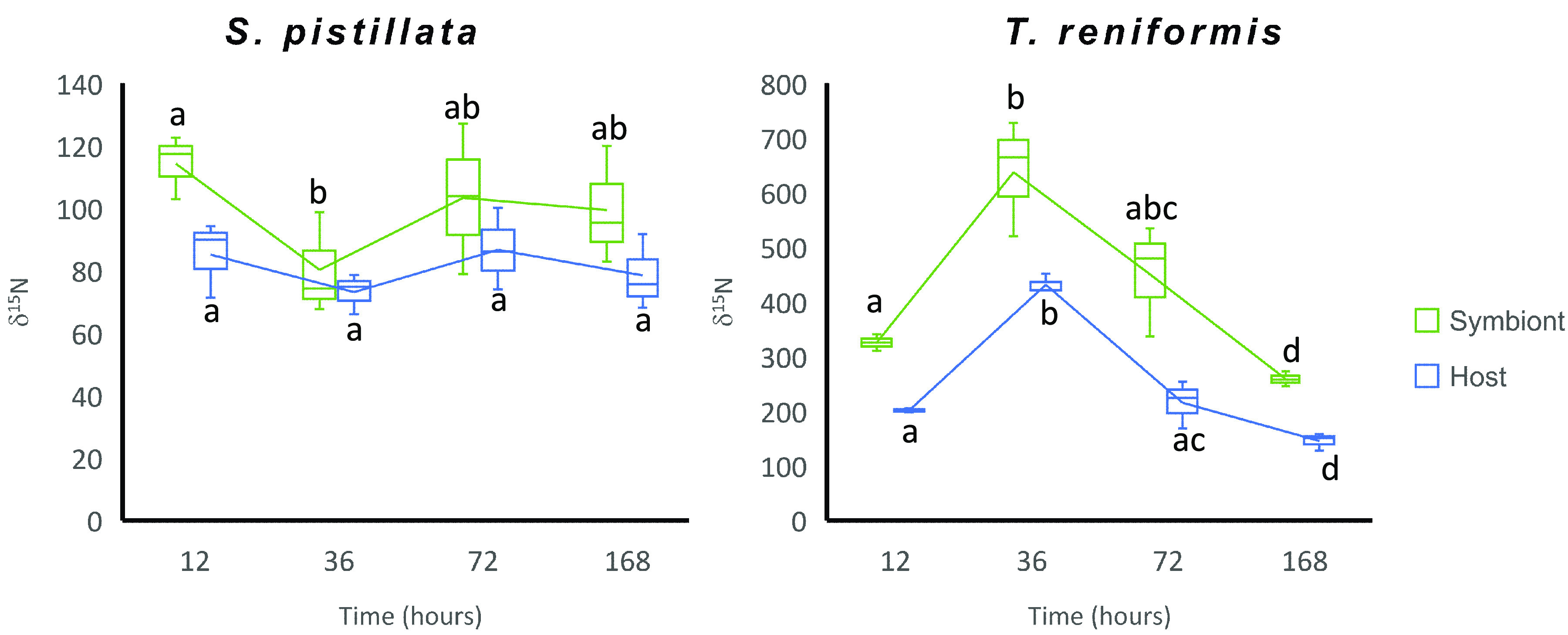
Pulse-chase experiment δ^15^N bulk signature of the host tissue (blue) and algal symbionts (green) of S. pistillata and T. reniformis fed during 6 h with ^15^N-labeled Artemia salina nauplii. Chase measurements were made 12, 36, 72, and 168 h after the pulse.

Overall, the A. salina nauplii AAs were highly enriched in ^15^N (Table 1). There was also a significant enrichment in *δ*^15^N of the tissue and symbionts of both S. pistillata and T. reniformis 12 h after being fed with ^15^N-labeled A. salina, confirming that both species can actively feed on plankton (Fig. 6 and Fig. S3). However, the enrichment was higher in T. reniformis than in S. pistillata, likely due to higher grazing rates or a larger amount of prey available because T. reniformis was lying on the bottom of the aquaria instead of being hung on a wire as for S. pistillata.

10.1128/mbio.01601-22.3 FIG S3 Pulse-chase experiment, comparison of the δ^15^N-AAs values of the host and symbionts of S. pistillata and T. reniformis fed during 6 h with ^15^N-labeled Artemia salina nauplii. Chase measurements were made 12, 36, 72, and 168 h after the pulse (dark to light grey, respectively). Amino acids with a white background were nonessential, and those with a grey background were essential AAs. Download FIG S3, EPS file, 0.6 MB. Copyright © 2022 Martinez et al. 2022 Martinez et al. https://creativecommons.org/licenses/by/4.0/ This content is distributed under the terms of the Creative Commons Attribution 4.0 International license.

For S. pistillata, there was no significant change in the host bulk *δ*^15^N value between the four sampling times (*P* > 0.18, Fig. 6). There was also no change in the *δ*^15^N for the symbionts between 12 h, 72 h, and 168 h (*P* > 0.24, Fig. 6), but there was a significantly lower *δ*^15^N at 36 h compared to 12 h (*P* = 0.04). For T. reniformis, the *δ*^15^N of both host and symbiont fractions showed a significant increase between 12 h and 36 h (*P* < 0.01 and *P* = 0.03, respectively, Fig. 6), followed by a continuous and significant decrease afterward (*P* < 0.044).

All amino acids of host and symbionts in both coral species were enriched in ^15^N acquired through the ingestion of *Artemia* nauplii (Fig. S3). However, the enrichment was significantly higher in the symbiont fraction (*P* < 0.01) and was observed after 12 h of incubation. In S. pistillata, no significant change in the *δ*^15^N-AAs could be observed during the chase in the host or symbiont compartments (*P* > 0.063). On the contrary, there was a reduction in T. reniformis *δ*^15^N enrichment of host and symbionts AAs over time with a significant difference between 12 and 168 h (*P* = 0.041 and *P* = 0.036, respectively).

## DISCUSSION

**FIG 7:**
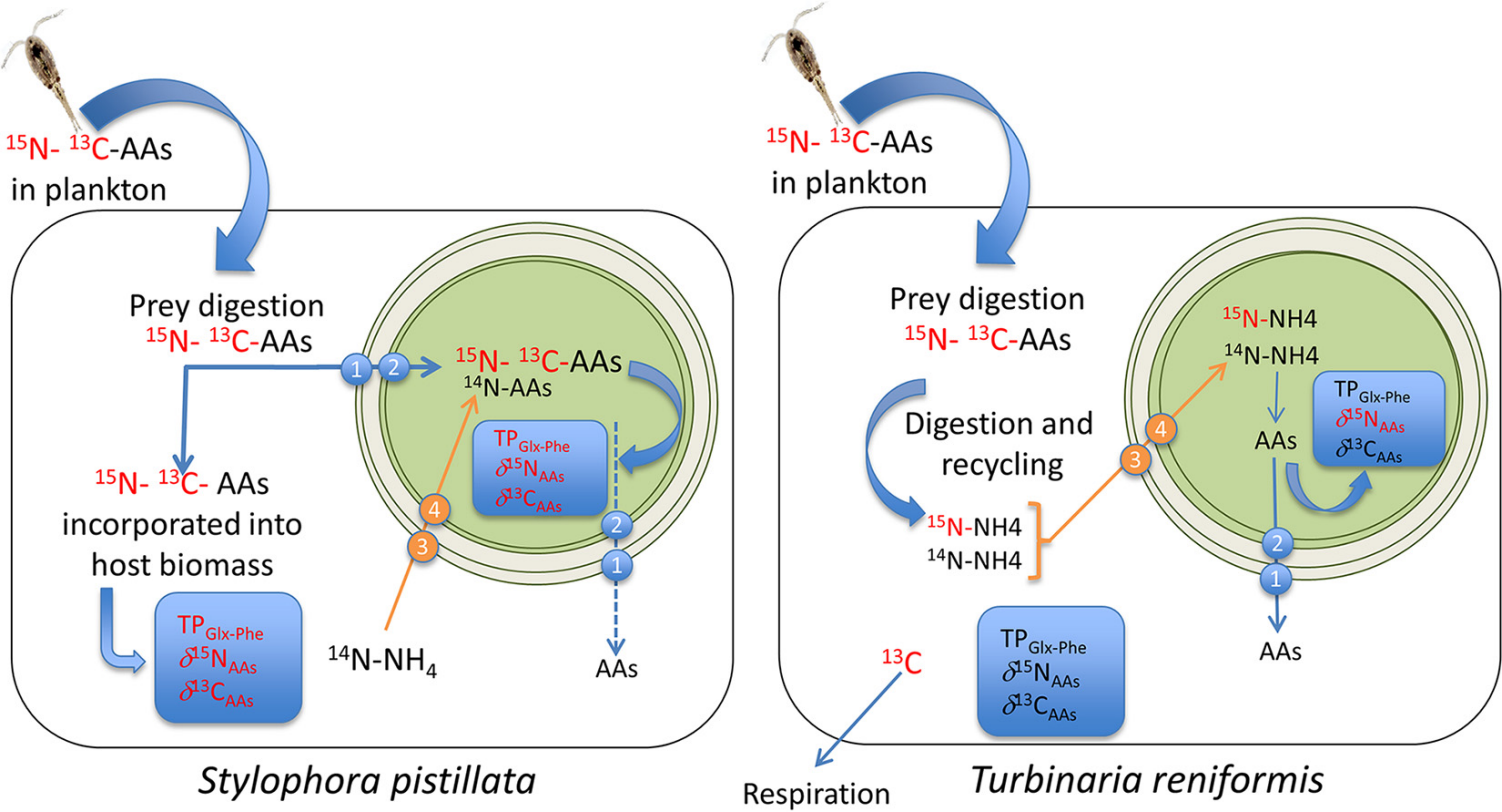
Schematic representation of the cycling of nutrients in the two symbiotic associations when fed Artemia salina prey. In S. pistillata, the significant increase (imaged in red color) in TP_Glx-Phe_, δ^15^N-AAs and δ^13^C-AAs in symbionts and host tissue with plankton feeding compared to unfed conditions indicatesd that the amino acids (AAs) were incorporated directly in host and symbiont biomass. AAs were transferred from host to symbiont through amino acid transporters (1, 2, 39, 40). In T. reniformis, the lack of change (imaged in black color) in TP_Glx-Phe_, and δ^13^C-AAs in symbionts with plankton feeding compared to unfed conditions indicated that the amino acids (AAs) were first recycled into ammonium (NH_4_), before being transferred via transporters (3, 4, 39, 40). to the symbionts and transformed into new AAs.

Estimation of the heterotrophic capacity of corals is key to predicting their resistance and resilience to climate change. Laboratory and *in situ* studies have repeatedly observed that bleaching and physiological impairment under stress are significantly lower in corals showing greater reliance on heterotrophy (14–18). Heterotrophic corals would, therefore, likely be among the winners of climate change. However, the importance of heterotrophy in the reef among corals is still largely unknown. Recent advances in CSIA-AA (*δ*^13^C*-δ*^15^N) open a new avenue to study trophic plasticity in reef-building corals but have yet to be validated for different coral-dinoflagellate associations. We have here combined this approach with isotopic prey labeling in unique controlled feeding experiments to quantitatively assess the flow of nutrients within the symbiotic association as well as the changes in *δ*^15^N and *δ*^13^C-AAs with the diet regime of corals. Therefore, this study provided an in-depth analysis of the effects of heterotrophy on the isotopic signatures of two coral holobionts, S. pistillata*-Symbiodinium* and T. reniformis*-Cladocopium*. We observed changes in the *δ*^15^N-AAs and trophic index of the symbionts of both coral species following heterotrophy, but the changes were species-dependent in the host (summarized in Fig. 7). Overall, the results suggested that the symbionts essentially benefited from the heterotrophically-acquired diet through translocation of nitrogen from their host and were vital components in the assimilation and transformation of the heterotrophic nutrients. However, while *Symbiodinium* sp. directly acquired heterotrophic amino acids, *Cladocopium* sp. rather received nitrogen from the waste products of the host catabolism. Baseline differences in the metabolism of the symbiont species could explain such differences.

The first objective of the study was to examine the changes in δ^13^C and δ^15^N-AAs levels (and TP_Glx-Phe)_ in host tissue when corals are fed a heterotrophic diet and investigate which coral host and symbiont directly assimilate dietary amino acids. Indeed, two previous controlled studies in which corals (Stylophora pistillata and Montipora capitata) were maintained under different trophic conditions yielded divergent results (35, 36). In addition, in this study, the changes in *δ*^13^C and *δ*^15^N-AAs were holobiont-dependent. While no effect of heterotrophy was detected in the tissue of T. reniformis, there was a significant change in all parameters (TP_Glx-Phe_, *δ*^13^C, and *δ*^15^N-AAs) and all amino acids (except valine and phenylalanine) of S. pistillata tissue upon heterotrophy. However, while δ^15^N-AAs and TP_Glx-Phe_ levels differed significantly between heterotrophic and autotrophic conditions, autotrophic and mixotrophic conditions, as found in the reef, could not be distinguished. Overall, these results, in conjunction with the previous studies, suggest that heterotrophy is more easily detected in the tissue of branching than massive species via CSIA-AAs, although more coral species and forms will need to be studied in the future to confirm this hypothesis.

The second objective of the study was to investigate whether translocation of dietary amino acids from the host to the symbionts occurs in one or the two symbiotic associations and, if so, which amino acids are preferentially translocated. In both coral species, heterotrophy increased the *δ*^15^N of all symbiont's AAs, regardless of the symbiont genotype, the host genotype, or the AA considered (essential, nonessential). This result suggested that symbionts use the heterotrophically acquired nitrogen for their own needs, while the host, especially T. reniformis, did not keep this nitrogen for itself. Even in complete darkness for several weeks, symbionts continue to assimilate heterotrophically derived nitrogen. As recently shown with Breviolum minutum strain SSB01 maintained in culture, direct supplementation of Symbiodiniaceae with amino acids can allow them to grow at a much higher rate and to maintain a more stable photosynthetic efficiency compared to when they are supplied with inorganic nutrients or seawater (39).

However, the TP_Glx-Phe_ of the symbionts, *δ*^15^N and *δ*^13^C-AAs, suggest a different pattern of nitrogen cycling within each symbiotic association. Indeed, while heterotrophy significantly increased the TP_Glx-Phe_ of both the host and symbionts of S. pistillata, it did not change the TP_Glx-Phe_ of T. reniformis. A TP_Glx-Phe_ of 1 indicates that the coral hosts or symbionts rely solely on the autotrophic supply of amino acids. In this case, the symbionts synthesize and translocate to the host new amino acids using inorganic nitrogen dissolved in seawater or recycled from host waste products (40), resulting in no fractionation. This was observed in symbionts of S. pistillata maintained under strict autotrophy. The significant increase in TP_Glx-Phe_ of S. pistillata symbionts under mixotrophy and heterotrophy (from 1 to 2) suggests that heterotrophically acquired nitrogen was shared with the symbionts by metabolic processes, resulting in ^15^N isotopic fractionation. These processes include transamination or oxidative deamination for protein synthesis and suggest a direct transfer of heterotrophically acquired AAs from S. pistillata host to symbionts via specific transporters (22, 41). Such transfer was confirmed by a significant and similar change in *δ*^15^N-AAs values of host and symbionts under mixotrophic and heterotrophic conditions (Fig. 7). Such direct nutrition of the symbionts by the coral host has been very recently highlighted by Jinkerson et al. (42). These authors showed that several coral species, which were able to provide the symbionts with food, hold and grow them regardless of their photosynthesis capabilities. Also, the significant change in *δ*^13^C-AAs in symbionts confirms that symbionts acquire entire AAs from the host diet. Similarly, in the pulse-chase experiment, the amino acids of S. pistillata host and symbionts were significantly enriched in ^15^N 12 h after the pulse of A. salina. Moreover, the enrichment did not change during 1 week, indicating that heterotrophic nitrogen, once acquired, was not used to meet metabolic needs but was assimilated into either storage or structural compounds (43).

In contrast to S. pistillata, the TP_Glx-Phe_ and *δ*^13^C-AAs of T. reniformis symbionts did not change with heterotrophy (Fig. 7), indicating that they did not directly assimilate heterotrophic AAs. However, the increase in symbiont *δ*^15^N-AAs suggests that the nitrogen acquired by heterotrophy was first recycled into ammonium before being transported to the symbiont (22, 41) and incorporated into symbionts' AAs. The pulse-chase experiment confirms this hypothesis. Bulk ^15^N enrichment indeed increased between 12 and 36 h, even though no ^15^N-labeled food was added to the system after the pulse. Such an increase suggests that prey was initially degraded into α-keto acids and ammonium, the latter compound being lost during tissue extraction and freeze-drying in the 12 h samples. In the 36 h samples, the increase in bulk ^15^N signal could be due to the reassimilation of ammonium into the biomass. A similar pattern was previously observed for both carbon and nitrogen but was ignored due to insignificant changes (3, 43, 44). After 36 h, the decrease in the *δ*^15^N enrichment of T. reniformis host and symbionts indicated either the catabolic excretion of labeled nitrogen or its dilution by newly absorbed ammonium. Such dilution of ^15^NH_4_ from the prey by newly incorporated ^14^NH_4_ may explain why in another experiment (results not published), no change in *δ*^15^N-AAs was observed in the symbionts following heterotrophic feeding of the host.

The results also showed that in S. pistillata, the host, and symbionts, regardless of nutritional conditions, used the same carbon source for the synthesis of their amino acids (no difference in *δ*^13^C-AAs of host and symbionts), as has been observed previously for many scleractinian species (11, 35–37). They also used the same nitrogen source, except in the autotrophic condition (5‰ difference in *δ*^15^N-AAs between host and symbionts). Under such conditions, the only nitrogen source for corals was inorganic nitrogen dissolved in seawater (DIN). Because DIN concentration was low under our culture conditions (<0.5 μM), corals may have suffered from nitrogen deficiency and starvation. The host might have gained the necessary nitrogen through symbiont phagocytosis (45). Therefore, the shift from one trophic position to the other might explain the higher *δ*^15^N-AAs values in all measured AAs of the host tissue. In contrast to S. pistillata, host and symbionts *δ*^13^C-AAs were different in T. reniformis, even in the autotrophic conditions where the only carbon source comes from symbiont photosynthates. Although this is not a general trend, Fujii et al. (38) obtained similar results in massive coral species. A relationship between symbiont density and host-symbiont differences in *δ*^13^C was also observed (46), with significant differences in corals with low symbiont density. Overall, these observations suggest that T. reniformis may have a completely different metabolism than S. pistillata and that further studies are needed to fully understand isotopic variations in the tissues of massive versus branching species.

The final aim of the study was to compare the isotopic values and TP_Glx-Phe_ of reef samples with values obtained with corals grown in aquaria. The reef corals exhibited higher *δ*^13^C-AAs signature than the aquarium-grown corals, likely due to a different light environment between the reef and the aquarium conditions. Aquaria was maintained at 200 ± 10 μmol photons m^−2^ s^−1^, corresponding to approximately 20 m (47), whereas reef samples were collected from 5m depth, where light is higher. Therefore, shallow reef corals exhibited higher *δ*^13^C-AAs isotopic signatures due to faster carbon assimilation rates (11). Reef samples also had lower *δ*^15^N-AAs than all aquarium treatments. Such a low isotopic ratio in corals from the Red Sea has been demonstrated previously (48, 49). It is probably due to nitrate with low *δ*^15^N values entering the Red Sea system in the form of aerosols (48, 50). The above observations suggest that *δ*^13^C and *δ*^15^N-AAs of coral tissue must be combined with plankton abundance and environmental data before they can be used as reliable proxies for heterotrophy. Although *δ*^13^C and *δ*^15^N-AAs values cannot be used to compare reef and aquarium-reared corals, the TP_Glx-Phe_ should be independent of the environmental isoscape (28, 30), and should reveal the trophic status of corals. Compared to aquarium conditions (AUT, MIX, and HET), the TP_Glx-Phe_ value of S. pistillata reef samples was similar to the mixotrophic and heterotrophic conditions, suggesting that this species should rely on a mixotrophic diet, even though the corals were collected at shallow depths, where autotrophy should provide most of their energetic requirements. This result is consistent with recent studies (12, 13, 38) and indicates that particle feeding is a major contributor to the energy budget of certain coral species. The TP_Glx-Phe_ value of reef samples of T. reniformis (1.2 for symbionts and 1.5 for host tissue), which was above 1, also suggested that this coral should rely on a mixotrophic diet. However, in aquaria, the TP_Glx-Phe_ value of T. reniformis did not increase with heterotrophy and had relatively high values (between 1.5 and 2) in the autotrophic condition. The inconsistency between heterotrophic capacities and TP_Glx-Phe_ observed in both *M. capitata* and T. reniformis (this study) could have several explanations. It could be due to a slow turnover of coral tissue associated with a low growth rate, suggesting that any dietary change requires several weeks/months before affecting TP_Glx-Phe_. Moreover, a previous study measured a lower change in skeletal growth rate in fed colonies of T. reniformis than in S. pistillata compared to unfed colonies (46). Fujii et al. (38) also found higher *δ*^15^N tissue values in massive than in branching species. In contrast, Hoogenboom et al. (46) observed an increase in *δ*^15^N with heterotrophy only in species where the contribution of heterotrophy to animal respiration was above 70%. While it was 70% in S. pistillata, it was less than 50% in T. reniformis. Overall, these results suggest that further studies on the informative value of TP_Glx-Phe_ of massive or foliose corals are needed to determine their heterotrophic stage.

The structural basis of coral reef ecosystems comes from the symbiosis between corals and Symbiodiniaceae. Such symbiosis is often described as the symbionts providing most of their photosynthates, including amino acids, to the coral host and, in exchange, receiving protection and host catabolic end products. The results obtained in this study showed a reverse translocation in which heterotrophically acquired nitrogen or amino acids were largely transferred from the coral hosts to their dinoflagellate symbionts. Furthermore, the TP_Glx-Phe_ of the coral reef samples suggests that the corals rely on a mixotrophic diet, even though they were collected at shallow depths where autotrophy should provide most of their energy needs. The translocation of heterotrophic nutrients, including metals, from the coral host to the Symbiodiniaceae is particularly important for maintaining symbiosis under environmental stress. This observation is of concern because plankton abundance in shallow reefs is likely to change under future climate conditions. Under the business-as-usual representative concentration pathway (RCP 8.5), phytoplankton and zooplankton biomass are predicted to decline by 6.1% and 13.6%, respectively (51). In addition, nutrient limitations will reduce the nitrogen and phosphorus content of phytoplankton cells relative to carbon content (51), meaning that planktivores will receive less food with lower nutritional value. Such changes in coral diets must be considered in current predictions of coral reefs.

## MATERIALS AND METHODS

### Biological material.

The experiments were performed with two scleractinian coral species abundant in the Northern Red Sea, Stylophora pistillata and Turbinaria reniformis. S. pistillata was a branching coral with minute polyps of ca. 0.8 mm in diameter, while T. reniformis was a laminar and foliose species with bigger polyps of 1.5 to 2 mm in diameter. Despite their difference in polyp size, both species are known to rely on heterotrophy for their diet (11), with T. reniformis being able to thrive in turbid and organically rich environments (52). Five colonies of each species were first collected under a special permit by the Israel Nature and Parks Authority from 5 m in front of the Interuniversity Institute for Marine Science (IUI), Gulf of Eilat, Northern Red Sea (29° 30′ N, 34° 56′ E). These colonies, subsequently designated as “reef samples”, were flash-frozen until subsequent analysis of CSIA-AAs (as described below) to determine their trophic status. The main Symbiodiniaceae genotype associated with each coral species was analyzed according to Santos et al. (53) protocol. As repeatedly observed (54, 55), S. pistillata was associated with *Symbiodinium* Clade A1, while T. reniformis was associated with *Cladocopium* clade C1.

In addition to the above samples, five mother colonies of each coral species were used in two laboratory experiments. Colonies were grown in controlled conditions in five open-water flow aquaria at the Centre Scientifique de Monaco. The temperature was constant at 25°C using heaters connected to Elli-Well PC 902/T controllers. Light (200 μM photons m^−2^ s^−1^) was provided with several HQI lamps. Light intensity was controlled by a LI-COR data logger (LI-1000) connected to a spherical quantum sensor (LI-193). Each mother colony was divided into several nubbins (see below) and they were allowed to heal for 3 to 4 weeks. During healing, they were fed once a week with Artemia salina nauplii to repletion.

### Trophic experiment.

For the first experiment, three large nubbins were generated from each of the five mother colonies and species (*n* = 15 per species). After healing, the nubbins were divided into three different nutritional treatments (heterotrophy, autotrophy, and mixotrophy), with one nubbin per colony and treatment. Each treatment included two aquaria, and nubbins were rotated between aquaria. All aquaria were continuously supplied with oligotrophic seawater pumped from 40 m and renewed at 12 L h^−1^. The water was filtered through sand filters and contained no large plankton prey. The temperature was kept at 25°C ± 0.2°C.

Corals in the heterotrophy treatment (HET) were maintained in the dark for 6 weeks, the maximal time before corals started to significantly bleach and then die. In this condition, corals did not receive any light but were fed to repletion five times a week with Artemia salina nauplii. The two other treatments, mixotrophy (MIX) and autotrophy (AUT) received an irradiance of 200 ± 10 μM photons m^−2^ s^−1^ (photoperiod was 12 h:12 h light:dark) provided by 400 W metal halide lamps (HPIT Philips). The mixotrophic corals were fed five times a week to repletion with A. salina nauplii, while the autotrophic corals were not fed. Corals were maintained in auto-or mixotrophy for 12 weeks. At the end of the incubation period, nubbins were sampled and immediately frozen at −80°C until subsequent analyses as described below.

### Pulse-chase experiment.

For this experiment, ^15^N-labeled A. salina nauplii were first prepared according to Tremblay et al. (9) (see Text S1). In addition, 12 nubbins were sampled from three colonies of each coral species (4 nubbins per colony). Each coral nubbin was placed in a 250 mL beaker on a magnetic stirrer and fed with one portion of A. salina for 5 h. After feeding, nubbins were kept in 4 aquaria for prey digestion and 3 nubbins from different colonies were sampled after 12, 36, 72, and 168 h. Nubbins were immediately frozen at −80°C until further analysis.

10.1128/mbio.01601-22.5 TEXT S1 Detailed explanation of the isotope in Materials and Methods. Download Text S1, DOCX file, 0.02 MB. Copyright © 2022 Martinez et al. 2022 Martinez et al. https://creativecommons.org/licenses/by/4.0/ This content is distributed under the terms of the Creative Commons Attribution 4.0 International license.

### Sample preparation.

For all nubbins, tissue was removed from the skeleton with an air pick, and the slurry was homogenized using a potter tissue grinder. The extracted slurry was centrifuged three times at 500 g for 10 min at 4°C to separate the host tissue from the symbionts. Afterward, the symbionts were washed twice with filtered seawater. The two fractions were freeze-dried until subsequent analysis of their bulk isotope and CSIA-AAs.

The nitrogen and carbon isotopic composition of amino acids was determined by gas chromatography/combustion/isotope ratio mass spectrometer (GC/C/IRMS) according to Martinez et al. (11) (see also Text S1). For the bulk isotopic measurements of the pulse-chase experiment, approximately 600 μg of lyophilized host and symbiont material were transferred to tin caps for analysis of the bulk isotopic ^15^N and ^13^C enrichment and the total carbon and nitrogen content using an Integra II isotope ratio mass spectrometer (Sercon, United Kingdom).

### Statistics.

Statistical analysis was done using PRIMER-e 7 with PERMANOVA+ add-on. Resemblance matrices were created for the data using the Euclidian distance followed by permutational multivariate analysis of variance (PERMANOVA) to test for statistical significance. When the number of repetitions was lower than 5 (pulse-chase experiment), we used PERMANOVA with Monte-Carlo. We also used PERMANOVA pairwise test to analyze significant differences between groups further. Only values with *P* < 0.05 were considered significant. Detailed statistic results can be found in Table S1.

10.1128/mbio.01601-22.4 TABLE S1 Results of statistical analyses using PRIMER 7 PERMANOVA. When *n* < 5 we used PERMANOVA with Monte-Carlo (MC). Comparisons were considered statistically significant where *P* < 0.05 and marked in bold. Download Table S1, DOCX file, 0.02 MB. Copyright © 2022 Martinez et al. 2022 Martinez et al. https://creativecommons.org/licenses/by/4.0/ This content is distributed under the terms of the Creative Commons Attribution 4.0 International license.
