# Supplementary material for: Symbiodiniaceae Are the First Site of Heterotrophic Nitrogen Assimilation in Reef-Building Corals
Source: mBio. 2022 Sep 20;13(5):e01601-22. doi: 10.1128/mbio.01601-22 (PMC9600528; doi:10.1128/mbio.01601-22)
Supplement: TEXT S1 [file mbio.01601-22-s0001.docx]

**Supplementary information**

**Symbiodiniaceae are the first site of heterotrophic nitrogen assimilation in reef-building corals**

Stephane Martinez^1^, Renaud Grover^1^, David M. Baker^2^ and Christine Ferrier-Pagès^1^

^1^Coral Ecophysiology Team, Centre Scientifique de Monaco, 8 Quai Antoine 1er, MC-98000 Monaco, Monaco

^2^The University of Hong Kong, The Swire Institute of Marine Science, School of Biological Sciences, Kadoorie Biological Sciences Building 6S-14, Pokfulam Road, Hong Kong, PRC

**Material and Methods**

Preparation of the ^15^N labelled artemia

The microalgae *Dunaliella* sp. was first grown in an f/2 enriched with 1 mmol L^−1^ of ^15^NH_4_Cl (98 atom %^15^N, cat. no. 299251, Sigma-Aldrich). Two-day-old nauplii were then fed on the ^15^N-labelled microalgae for four days. *A. salina* were isolated by filtration on a 20 µm mesh, divided into equal portions, and frozen at −20°C. Each portion corresponded to 22.9 ± 1.2 mg dry weight.

Amino acid stable isotope analysis

Acid hydrolyzed host and symbiont samples were derivatized prior to the isotope analysis using the Ezfaast kit following Martinez et al. (2020) methods. Briefly, approximately 3.5 mg hydrolyzed samples underwent derivatization using the Ezfaast kit with a slight modification of replacing reagent 6 with dichloromethane as a solvent. The amino acids were separated on a Zebron ZB-50 column (30 m, 0.25 mm, and 0.25 µm) on a Thermo Scientific Trace 1300 Gas Chromatograph with helium as a carrier gas at a constant flow of 1.5 ml/min. For carbon analysis, 1.5 µl was injected in split mode (1:15) at 250°C and 2 µl was injected in split mode (1:5) at 250°C for nitrogen analysis. The separated amino acids were split on the MicroChannel device into two direction flows: Thermo Scientific ISQ quadruple for amino acid identification and Thermo Scientific Delta-V advantage for C and N isotope analysis. To define the isotopic ratio of carbon and nitrogen the separated amino acids were combusted in a Thermo scientific GC isolink II at 1000 °C for CO_2_ and N_2_. Before entering Delta-V for the N_2_ analysis, the sample went through a liquid nitrogen cold trap to freeze all other gases. From each sample, duplicates were injected for carbon and triplicates for nitrogen. Stable isotope ratios were expressed in standard δ notation where the standard for carbon was Vienna PeeDee Belemnite (VPDB) and for nitrogen atmospheric N_2_ (air). To account for the carbons incorporated during the derivatization process, we followed Docherty et al. (2001) correction factor for each amino acid. The trophic position (TP_Glx-Phe_) was calculated using glutamic acid and phenylalanine with the predefined equation from Chikaraishi et al. (2009) with the constants from Martinez et al. (2020).

**References**

Chikaraishi, Y., Ogawa, N. O., Kashiyama, Y., Takano, Y., Suga, H., Tomitani, A., et al. (2009). Determination of aquatic food-web structure based on compound-specific nitrogen isotopic composition of amino acids. *Limnol. Oceanogr. Methodes* 7, 740–750. Available at: https://www.aslo.org/lomethods/free/2009/0740.pdf [Accessed June 11, 2014].

Docherty, G., Jones, V., and Evershed, R. P. (2001). Practical and theoretical considerations in the gas chromatography/combustion/isotope ratio mass spectrometry delta(13)C analysis of small polyfunctional compounds. *Rapid Commun. Mass Spectrom.* 15, 730–8. doi:10.1002/rcm.270.

Martinez, S., Lalzar, M., Shemesh, E., Einbinder, S., Goodman, B., and Tchernov, D. (2020). Effect of different derivatization protocols on the calculation of trophic position using amino acids compound-specific stable isotopes. *Front. Mar. Sci.* 7, 1–7. doi:10.3389/fmars.2020.561568.
