# Supplementary material for: Symbiodiniaceae Are the First Site of Heterotrophic Nitrogen Assimilation in Reef-Building Corals
Source: mBio. 2022 Sep 20;13(5):e01601-22. doi: 10.1128/mbio.01601-22 (PMC9600528; doi:10.1128/mbio.01601-22)
Supplement: TABLE S1 [file mbio.01601-22-s0002.docx]

**Table S1** Results of statistical analyses using PRIMER 7 PERMANOVA. When n<5 we used PERMANOVA with monte-carlo (MC). Comparisons are considered statistically significant where p = <0.05 and marked in bold.

| **Parameter** | **Method** | **t** | ***p* value** | **unique perms** |
| --- | --- | --- | --- | --- |
| **Natural AA-CSIA carbon** |  |  |  |  |
| *S.pisttilata* host symbiont | PERMANOVA |  | 0.668 | 998 |
| *S.pisttilata* host | PERMANOVA |  | **0.001** | 999 |
| AUT, MIX | PERMANOVA | 1.4721 | 0.098 | 126 |
| AUT, HET | PERMANOVA | 1.7027 | **0.026** | 126 |
| AUT, Reef | PERMANOVA | 1.9425 | **0.031** | 411 |
| MIX, HET | PERMANOVA | 1.5607 | 0.136 | 126 |
| MIX, Reef | PERMANOVA | 2.7183 | **0.005** | 399 |
| HET, Reef | PERMANOVA | 2.4378 | **0.004** | 411 |
| *S.pisttilata* symbiont | PERMANOVA |  | **0.001** | 997 |
| AUT, MIX | PERMANOVA | 2.222 | **0.034** | 126 |
| AUT, HET | PERMANOVA | 2.1774 | **0.01** | 126 |
| AUT, Reef | PERMANOVA | 2.3235 | **0.019** | 126 |
| MIX, HET | PERMANOVA | 1.515 | 0.079 | 126 |
| MIX, Reef | PERMANOVA | 3.3757 | **0.008** | 126 |
| HET, Reef | PERMANOVA | 3.1283 | **0.007** | 125 |
| *T. reinformis* host symbiont | PERMANOVA |  | **0.003** | 998 |
| HET | PERMANOVA | 1.0014 | 0.461 | 126 |
| MIX | PERMANOVA | 1.9522 | **0.042** | 126 |
| AUT | PERMANOVA | 2.118 | **0.036** | 21 |
| Reef | PERMANOVA | 0.86699 | 0.515 | 126 |
| *T. reinformis* host | PERMANOVA |  | **0.001** | 994 |
| HET, MIX | PERMANOVA | 1.7369 | 0.096 | 35 |
| HET, AUT | PERMANOVA | 1.2332 | 0.263 | 15 |
| HET, Reef | PERMANOVA | 3.777 | **0.03** | 35 |
| MIX, AUT | PERMANOVA | 0.62488 | 0.868 | 15 |
| MIX, Reef | PERMANOVA | 2.5812 | **0.025** | 35 |
| AUT, Reef | PERMANOVA | 2.0603 | 0.072 | 15 |
| *T. reinformis* symbiont | PERMANOVA |  | **0.001** | 998 |
| HET, MIX | PERMANOVA | 1.2444 | 0.248 | 126 |
| HET, AUT | PERMANOVA | 1.155 | 0.29 | 126 |
| HET, Reef | PERMANOVA | 5.6345 | **0.009** | 126 |
| MIX, AUT | PERMANOVA | 1.1584 | 0.286 | 126 |
| MIX, Reef | PERMANOVA | 4.9069 | **0.01** | 126 |
| AUT, Reef | PERMANOVA | 5.7954 | **0.006** | 126 |
|  |  |  |  |  |
| **Natural AA-CSIA nitrogen** |  |  |  |  |
| *S.pisttilata* host symbiont | PERMANOVA |  | **0.001** | 999 |
| HET | PERMANOVA | 0.73417 | 0.716 | 126 |
| MIX | PERMANOVA | 1.1408 | 0.245 | 126 |
| AUT | PERMANOVA | 5.1606 | **0.028** | 35 |
| Reef | PERMANOVA | 2.1152 | **0.052** | 126 |
| *S.pisttilata* host | PERMANOVA |  | **0.001** | 997 |
| HET, AUT | PERMANOVA | 2.4051 | **0.017** | 126 |
| HET, MIX | PERMANOVA | 2.5839 | **0.01** | 126 |
| HET, Reef | PERMANOVA | 6.7003 | **0.005** | 126 |
| AUT, MIX | PERMANOVA | 1.1603 | 0.251 | 126 |
| AUT, Reef | PERMANOVA | 6.5339 | **0.01** | 126 |
| MIX, Reef | PERMANOVA | 6.4447 | **0.008** | 126 |
| *S.pisttilata* symbiont | PERMANOVA |  | **0.001** | 999 |
| HET, AUT | PERMANOVA | 6.1629 | **0.035** | 35 |
| HET, MIX | PERMANOVA | 3.3591 | **0.012** | 126 |
| HET, Reef | PERMANOVA | 8.6017 | **0.01** | 126 |
| AUT, MIX | PERMANOVA | 3.7289 | **0.01** | 126 |
| AUT, Reef | PERMANOVA | 3.3865 | **0.017** | 126 |
| MIX, Reef | PERMANOVA | 6.6843 | **0.01** | 126 |
| *T. reinformis* host symbiont | PERMANOVA |  | **0.001** | 999 |
| HET | PERMANOVA | 4.3754 | **0.011** | 126 |
| MIX | PERMANOVA | 2.5415 | **0.009** | 404 |
| AUT | PERMANOVA | 1.6663 | 0.086 | 56 |
| Reef | PERMANOVA | 0.88535 | 0.509 | 56 |
| *T. reinformis* host | PERMANOVA |  | **0.002** | 998 |
| HET, MIX | PERMANOVA | 0.97923 | 0.449 | 208 |
| HET, AUT | PERMANOVA | 0.91087 | 0.4 | 35 |
| HET, Reef | PERMANOVA | 4.3302 | **0.029** | 35 |
| MIX, AUT | PERMANOVA | 0.87156 | 0.453 | 84 |
| MIX, Reef | PERMANOVA | 4.6571 | **0.017** | 84 |
| AUT, Reef | PERMANOVA | 2.7702 | **0.027** | 10 |
| *T. reinformis* symbiont | PERMANOVA |  | **0.001** | 998 |
| HET, MIX | PERMANOVA | 2.3887 | **0.01** | 126 |
| HET, AUT | PERMANOVA | 4.6179 | **0.011** | 126 |
| HET, Reef | PERMANOVA | 14.128 | **0.009** | 126 |
| MIX, AUT | PERMANOVA | 2.3873 | **0.016** | 126 |
| MIX, Reef | PERMANOVA | 10.582 | **0.007** | 126 |
| AUT, Reef | PERMANOVA | 7.7913 | **0.007** | 126 |
|  |  |  |  |  |
| **Trophic position** |  |  |  |  |
| *S.pisttilata* host | PERMANOVA |  | **0.001** | 999 |
| HET, AUT | PERMANOVA | 2.4034 | **0.024** | 118 |
| HET, MIX | PERMANOVA | 2.0033 | 0.067 | 126 |
| HET, Reef | PERMANOVA | 1.2346 | 0.244 | 126 |
| AUT, MIX | PERMANOVA | 1.4867 | 0.2 | 126 |
| AUT, Reef | PERMANOVA | 3.6504 | **0.019** | 126 |
| MIX, Reef | PERMANOVA | 1.8412 | 0.108 | 126 |
| *S.pisttilata* symbiont | PERMANOVA |  | **0.001** | 999 |
| HET, AUT | PERMANOVA | 4.2491 | **0.028** | 35 |
| HET, MIX | PERMANOVA | 3.2385 | **0.016** | 126 |
| HET, Reef | PERMANOVA | 3.483 | **0.02** | 126 |
| AUT, MIX | PERMANOVA | 2.8852 | **0.029** | 126 |
| AUT, Reef | PERMANOVA | 1.9371 | 0.097 | 126 |
| MIX, Reef | PERMANOVA | 0.67905 | 0.528 | 126 |
| *T. reinformis* host | PERMANOVA |  | 0.482 | 999 |
| HET, MIX | PERMANOVA | 0.57345 | 0.567 | 209 |
| HET, AUT | PERMANOVA | 0.48162 | 0.649 | 35 |
| HET, Reef | PERMANOVA | 0.79694 | 0.436 | 35 |
| MIX, AUT | PERMANOVA | 1.1452 | 0.349 | 84 |
| MIX, Reef | PERMANOVA | 1.4477 | 0.184 | 84 |
| AUT, Reef | PERMANOVA | 0.37304 | 0.619 | 10 |
| *T. reinformis* symbiont | PERMANOVA |  | **0.001** | 998 |
| HET, MIX | PERMANOVA | 2.4744 | **0.041** | 126 |
| HET, AUT | PERMANOVA | 1.7828 | 0.123 | 126 |
| HET, Reef | PERMANOVA | 7.735 | **0.009** | 126 |
| MIX, AUT | PERMANOVA | 0.84974 | 0.411 | 126 |
| MIX, Reef | PERMANOVA | 3.6275 | **0.008** | 125 |
| AUT, Reef | PERMANOVA | 5.4694 | **0.005** | 126 |
|  |  |  |  |  |
| **SIP bulk nitrogen** |  |  |  |  |
| *S.pisttilata* host |  |  |  |  |
| 12Hour, 36Hour | (MC) PERMANOVA | 1.5221 | 0.201 | 10 |
| 12Hour, 72Hour | (MC) PERMANOVA | 0.14661 | 0.892 | 10 |
| 12Hour, 168Hour | (MC) PERMANOVA | 0.68245 | 0.528 | 10 |
| 36Hour, 72Hour | (MC) PERMANOVA | 1.6141 | 0.183 | 10 |
| 36Hour, 168Hour | (MC) PERMANOVA | 0.68124 | 0.541 | 10 |
| 72Hour, 168Hour | (MC) PERMANOVA | 0.8037 | 0.455 | 10 |
| *S.pisttilata* symbiont |  |  |  |  |
| 12Hour, 36Hour | (MC) PERMANOVA | 3.0654 | **0.051** | 10 |
| 12Hour, 72Hour | (MC) PERMANOVA | 0.72194 | 0.516 | 10 |
| 12Hour, 168Hour | (MC) PERMANOVA | 1.1957 | 0.294 | 10 |
| 36Hour, 72Hour | (MC) PERMANOVA | 1.3785 | 0.239 | 10 |
| 36Hour, 168Hour | (MC) PERMANOVA | 1.3345 | 0.26 | 10 |
| 72Hour, 168Hour | (MC) PERMANOVA | 0.22199 | 0.831 | 10 |
| *T. reinformis* host |  |  |  |  |
| 12Hour, 36Hour | (MC) PERMANOVA | 17.037 | **0.002** | 10 |
| 12Hour, 72Hour | (MC) PERMANOVA | 0.43366 | 0.698 | 10 |
| 12Hour, 168Hour | (MC) PERMANOVA | 4.5783 | **0.018** | 10 |
| 36Hour, 72Hour | (MC) PERMANOVA | 7.9625 | **0.003** | 10 |
| 36Hour, 168Hour | (MC) PERMANOVA | 20.788 | **0.001** | 10 |
| 72Hour, 168Hour | (MC) PERMANOVA | 2.6179 | 0.044 | 10 |
| *T. reinformis* symbiont |  |  |  |  |
| 12Hour, 36Hour | (MC) PERMANOVA | 3.9122 | **0.03** | 10 |
| 12Hour, 72Hour | (MC) PERMANOVA | 1.6253 | 0.206 | 10 |
| 12Hour, 168Hour | (MC) PERMANOVA | 4.4585 | **0.018** | 10 |
| 36Hour, 72Hour | (MC) PERMANOVA | 2.2031 | 0.083 | 10 |
| 36Hour, 168Hour | (MC) PERMANOVA | 6.1391 | **0.007** | 10 |
| 72Hour, 168Hour | (MC) PERMANOVA | 3.2226 | **0.021** | 10 |
|  |  |  |  |  |
| **SIP AA-CSIA nitrogen** |  |  |  |  |
| *S.pisttilata* host |  |  |  |  |
| 12Hour, 36Hour | (MC) PERMANOVA | 1.4691 | 0.206 | 10 |
| 12Hour, 72Hour | (MC) PERMANOVA | 1.2538 | 0.262 | 10 |
| 12Hour, 168Hour | (MC) PERMANOVA | 0.99291 | 0.392 | 10 |
| 36Hour, 72Hour | (MC) PERMANOVA | 0.42647 | 0.751 | 10 |
| 36Hour, 168Hour | (MC) PERMANOVA | 0.75989 | 0.525 | 10 |
| 72Hour, 168Hour | (MC) PERMANOVA | 0.48648 | 0.701 | 10 |
| *S.pisttilata* symbiont |  |  |  |  |
| 12Hour, 36Hour | (MC) PERMANOVA | 2.3317 | 0.063 | 10 |
| 12Hour, 72Hour | (MC) PERMANOVA | 1.2446 | 0.267 | 10 |
| 12Hour, 168Hour | (MC) PERMANOVA | 0.80047 | 0.464 | 10 |
| 36Hour, 72Hour | (MC) PERMANOVA | 0.90566 | 0.423 | 10 |
| 36Hour, 168Hour | (MC) PERMANOVA | 1.894 | 0.114 | 10 |
| 72Hour, 168Hour | (MC) PERMANOVA | 0.7072 | 0.491 | 10 |
| *T. reinformis* host |  |  |  |  |
| 12Hour, 72Hour | (MC) PERMANOVA | 1.603 | 0.169 | 10 |
| 12Hour, 36Hour | (MC) PERMANOVA | 0.65228 | 0.606 | 10 |
| 12Hour, 168Hour | (MC) PERMANOVA | 2.7792 | **0.041** | 10 |
| 72Hour, 36Hour | (MC) PERMANOVA | 1.0543 | 0.357 | 10 |
| 72Hour, 168Hour | (MC) PERMANOVA | 1.2445 | 0.262 | 10 |
| 36Hour, 168Hour | (MC) PERMANOVA | 2.1473 | 0.086 | 10 |
| *T. reinformis* symbiont |  |  |  |  |
| 12Hour, 72Hour | (MC) PERMANOVA | 0.7373 | 0.499 | 10 |
| 12Hour, 36Hour | (MC) PERMANOVA | 0.19441 | 0.91 | 10 |
| 12Hour, 168Hour | (MC) PERMANOVA | 2.9842 | **0.036** | 10 |
| 72Hour, 36Hour | (MC) PERMANOVA | 0.47688 | 0.665 | 10 |
| 72Hour, 168Hour | (MC) PERMANOVA | 1.6067 | 0.195 | 10 |
| 36Hour, 168Hour | (MC) PERMANOVA | 2.0327 | 0.101 | 10 |
